# Case Report: Modified surgery combined with photodynamic therapy for treating basal cell carcinoma in special facial regions

**DOI:** 10.3389/fonc.2025.1670270

**Published:** 2025-09-18

**Authors:** Zhixia Fan, Chao Lv, Panpan Suo, Lei Shi, Guoying Miao

**Affiliations:** ^1^ Department of Dermatology, Hebei Engineering University Affiliated Hospital, Handan, Hebei,, China; ^2^ Department of Dermatology, Huadong Hospital, Fudan University, Shanghai, China

**Keywords:** basal cell carcinoma, photodynamic therapy (PDT), regional cancerous tissue, modified surgery, special facial regions

## Abstract

Basal cell carcinoma (BCC) is one of the most common malignant skin tumors, predominantly occurring in sun-exposed areas such as the head and face. The combination of surgery and photodynamic therapy (PDT) enables rapid tumor clearance and comprehensive regional treatment of cancerous tissues, effectively reducing recurrence rates. This report presents two cases of patients with basal cell carcinoma in special facial areas treated with modified surgical excision combined with photodynamic therapy.

## Case report

### Case 1

A 50-year-old female patient. Eight years ago, a rash appeared in front of the right tragus and gradually enlarged, with surface ulceration after scratching. An irregular plaque measuring 1.9cm*1.7cm was observed on the right tragus, exhibiting pearl-like raised edges and central ulceration, with some lesions extending across the tragus to the external auditory canal.

pathological biopsy: Tumor cell nests in the superficial dermis with peripheral cells arranged in a palisading pattern, and retraction spaces visible at the margins.

reflectance confocal microscopy: Spoke-wheel arrangement of tumor masses with high-refractive-index pigments within the tumor nests.

**Figure 1:**
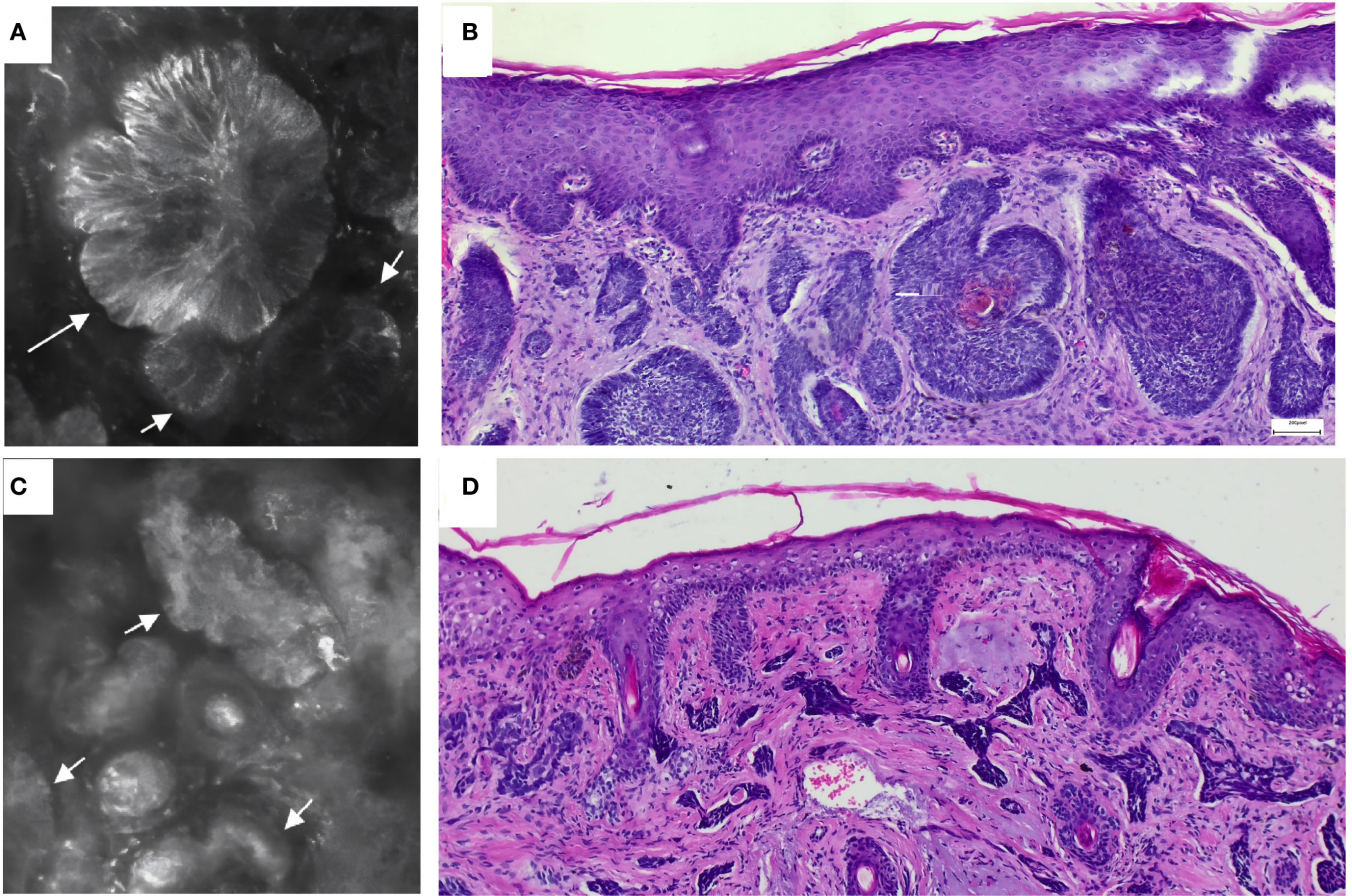
**(A, C)** reflectance confocal microscopy show that the high-refractive area indicated by the arrow is a tumor mass. **(B)** Histopathology of case 1 shows reveals a mass-like cellular tumor with peripheral cells arranged in a palisade pattern. **(D)** Histopathology of case 2 shows a cord-like cellular tumor mass.

Diagnosis: Nodular basal cell carcinoma (**Figures 1A, B**).

### Case 2

**A** 69-year-old female patient developed gradually enlarging rashes one year ago. Currently, two adjacent erythematous patches approximately the size of mung beans (about 1cm*0.9cm) are visible on the nasal tip, with moist surfaces and a small amount of serous crusts.

pathological biopsy: Dermal nodular or cord-like tumor cell nests, interstitial collagen proliferation, and mucin deposition.

reflectance confocal microscopy: Cord-like or nodular tumor masses with cleft formation in the surrounding tissue.

Diagnosis: morpheaform basal cell carcinoma. (**Figures 1C, D**).

### Pretreatment evaluation

Referring to the U.S. “BCC Clinical Practice Guidelines Version 2024.2” and Chinese clinical practice, risk assessments were conducted for both patients: both patients were diagnosed with high-risk BCC (1, 2).

### Treatment course

The first-line treatment for high-risk BCC is surgical excision. Considering the size and anatomical complexity of the lesions in both patients, we performed modified surgical excision (with 0.5cm margins). In Patient 1, a skin flap was used to cover the postoperative wound in the preauricular area, while the ear canal wound in Patient 1 and the nasal surgical wound in Patient 2 were managed with laser hemostasis and left for secondary healing.

Both patients started photodynamic therapy one week after surgery and completed the treatment within a month. Patient 1 received three sessions of photodynamic therapy, while patient 2 received four sessions of photodynamic therapy, with a one-week interval between each session. Each session took about four hours.

The photodynamic therapy procedure is as follows: Sterile gauze soaked in freshly prepared 20%ALA solution is applied to cover the lesion and its surrounding 0.5-1cm area for enhanced safety margins. After 3 hours of light-shielding application, red light irradiation (LED with 80 J/cm² energy density) was administered for 20 minutes on both patient 1’s anterior ear and patient 2’s nasal region. Additionally, patient 1 received 20 minutes of red light fiber irradiation in the ear canal at the same energy density. Fiber optic irradiation was chosen as it provides superior coverage of uneven ear areas.

**Figure 2:**
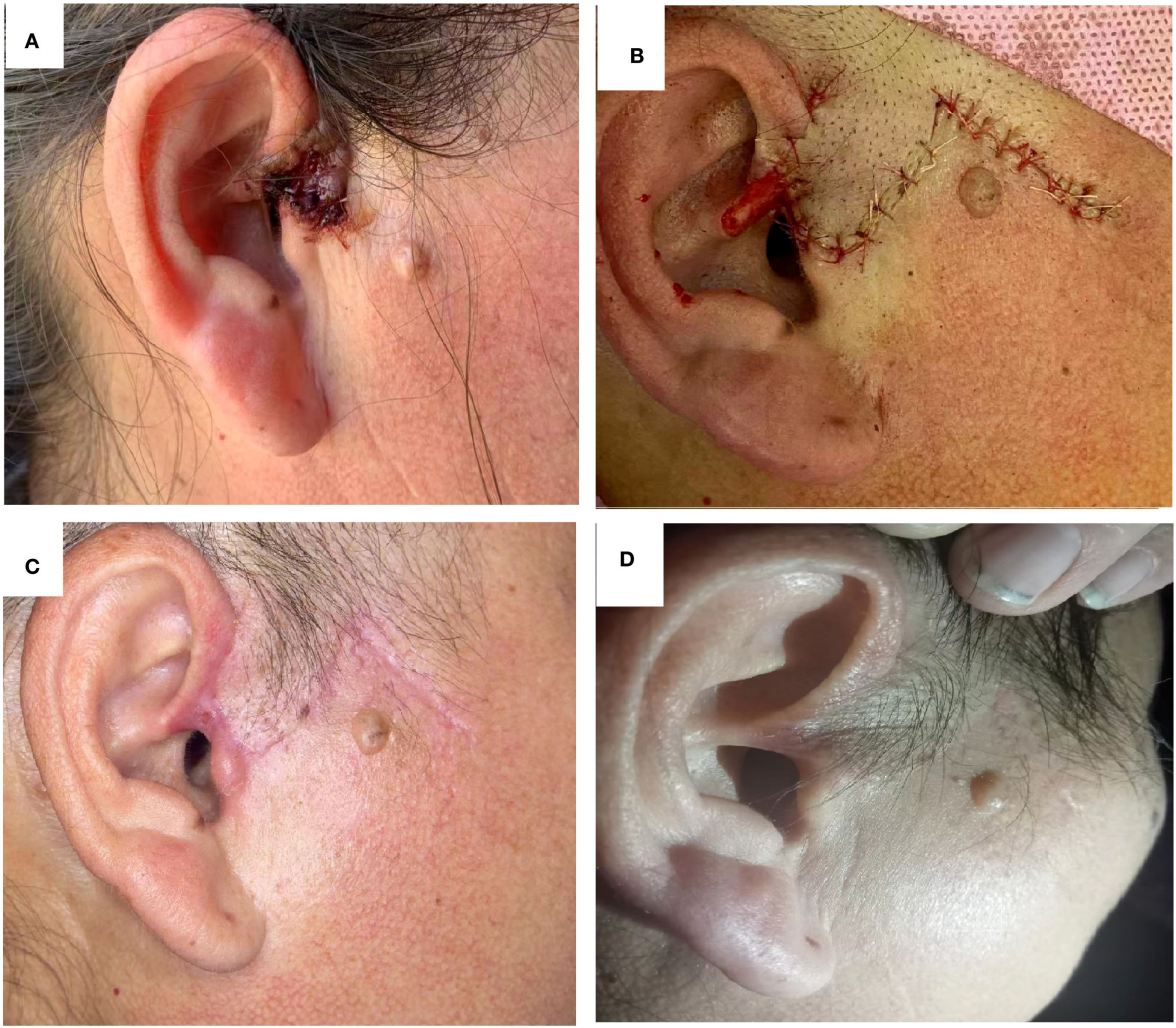
Case 1 Treatment course **(A)** image of lesion before treatment; **(B)** Postoperative surgical excision; **(C)** After three photodynamic therapy sessions; **(D)** Two-year follow-up.

**Figure 3:**
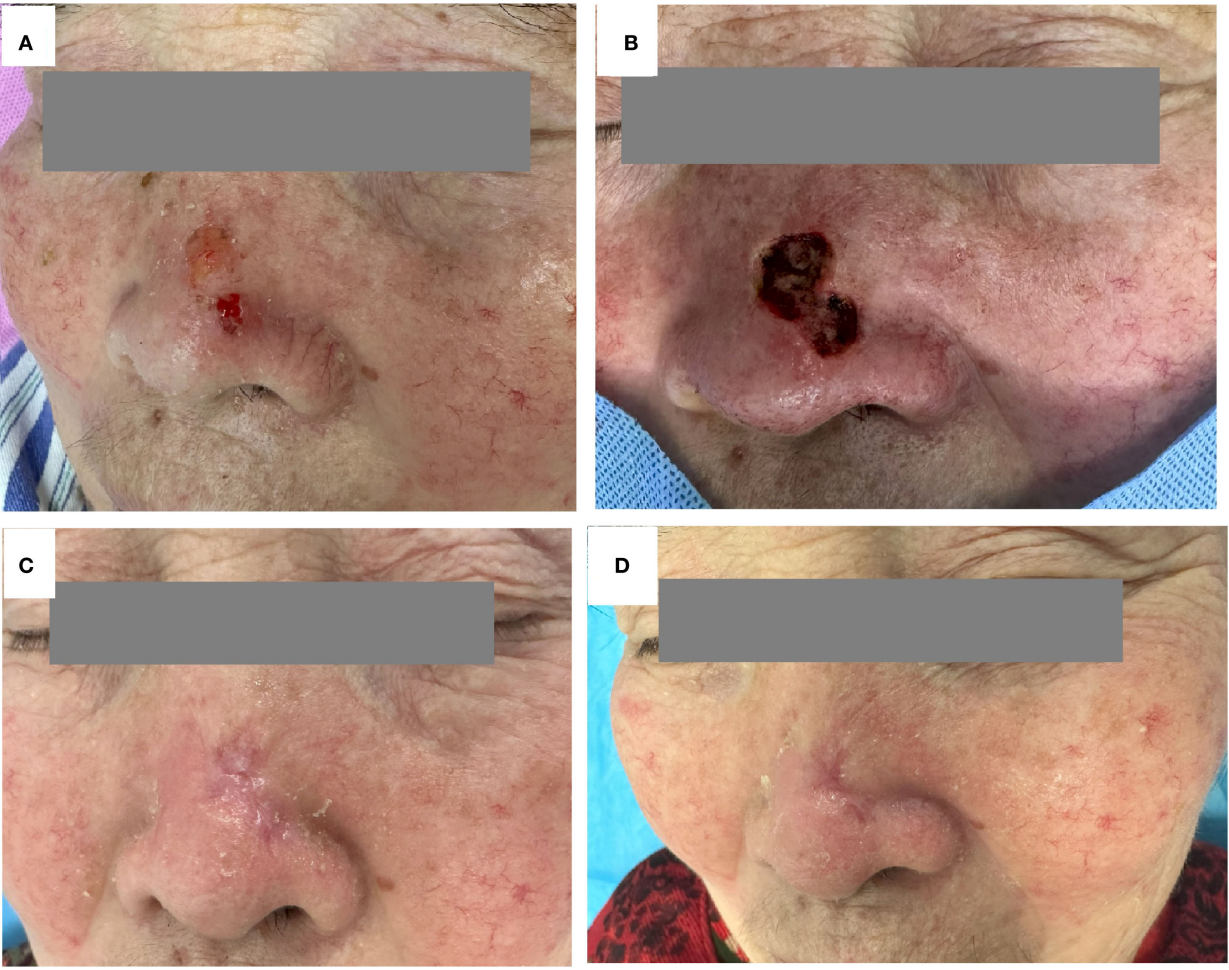
Case 2 Treatment Course: **(A)** image of lesion before treatment; **(B)** Postoperative status after surgical excision; **(C)** After four sessions of photodynamic therapy; **(D)** One-year follow-up.

During treatment, both patients experienced only mild redness, swelling, pain, and a burning sensation in the treated area with slight exudation, without other discomforts. After symptomatic treatment, all symptoms gradually subsided. The two patients underwent 1-2 years of follow-up without recurrence, showing good skin lesion recovery and no significant scar formation. (**Figures 2**, **3**).

## Discussion

Basal cell carcinoma of the skin grows relatively slowly, and early diagnosis and treatment can reduce therapeutic difficulty and cost (3–6). histopathology is the gold standard for diagnosis, while detection tools such as.

reflectance confocal microscopy enable early differential diagnosis (7, 8). To avoid overtreatment or undertreatment, both patients underwent risk assessment and received modified resection combined with postoperative adjuvant photodynamic therapy. The modified surgical procedure (extended excision of 0.5cm) removed clinically visible tumor masses while maximally preserving nasal and auricular appearance and function. However, statistics show that the incomplete resection rate for high-risk BCC after extended excision remains between 3.2%-61.5% (9). Postoperative treatment of “regional cancerous tissue” is key to reducing recurrence rates, and photodynamic therapy clearly represents the optimal choice.

Photodynamic therapy can selectively target and kill tumor cells, induce immune responses, and inhibit the progression of regional microenvironmental carcinogenesis. Compared to surgical resection (local treatment), PDT can more thoroughly eliminate occult lesions (10), achieving “regional carcinogenesis tissue therapy” with high tumor clearance rates and low recurrence rates. A retrospective clinical study comparing surgery combined with photodynamic therapy (S-PDT) versus Mohs micrographic surgery (MMS) for refractory basal cell carcinoma found no statistically significant difference in recurrence rates between S-PDT and MMS (P = 1.000), with a median follow-up time of 42.5 months (range: 36-63 months) (11). Photodynamic therapy can also serve as a preoperative neoadjuvant therapy, where preoperative PDT significantly reduces tumor size, increases surgical feasibility, and lowers the risk of tumor recurrence (12).

The two cases share the common characteristic of having lesions in special areas (facial H-zone/ears), which are classified as high-risk BCC. Modified surgery can rapidly remove the tumor mass but fails to completely eliminate the “regional cancerous tissue” at the tumor margins. PDT is more adept at treating superficial diffuse cancerous tissue and activating the body’s immune system. However, due to the limited penetration of photosensitizers and light sources, standalone PDT shows suboptimal clinical efficacy for large or deep BCC tumors. Based on this, we combined both methods and achieved satisfactory clinical outcomes, with no recurrence observed during the 1-2 year follow-up for both patients. Additionally, excellent cosmetic results were noted, with no significant scarring at the lesion sites, leading to improved quality of life for the patients.

This suggests that selecting an appropriate surgical plan combined with photodynamic therapy for BCC patients with lesions in special locations, multiple tumors, or inability to tolerate complex surgery is one of the effective approaches to improve cure rates and reduce recurrence rates. This combined approach not only minimizes structural and functional damage caused by surgical trauma but also utilizes PDT to comprehensively eliminate residual tumor cells, achieving favorable clinical outcomes. It provides meaningful and innovative references for clinicians encountering similar cases in the future.

The two cases reported in this study are limited by a small sample size, which affects the generalizability of our conclusions. Future research should validate these findings through large-scale cohort studies with longer follow-up periods (>5 years). Additionally, randomized controlled trials (surgical alone VS surgery and photodynamic therapy) should be conducted to comprehensively evaluate the clinical efficacy and recurrence rates of these two treatment approaches.

## Data Availability

The original contributions presented in the study are included in the article/supplementary material. Further inquiries can be directed to the corresponding author.
